# The Ultrashort Spike–Ring Interaction in Substituted Iron Maiden Molecules

**DOI:** 10.3390/molecules28052244

**Published:** 2023-02-28

**Authors:** Mirosław Jabłoński

**Affiliations:** Faculty of Chemistry, Nicolaus Copernicus University in Toruń, Gagarina 7, 87-100 Toruń, Poland; teojab@chem.umk.pl; Tel.: +48-056-611-4695

**Keywords:** ultrashort interaction, iron maiden, cage, endohedral complex, repulsion, hindrance, steric effects, substituent, substituent effects, electronic effects

## Abstract

The *in* forms of molecular iron maidens are known for their unique ultrashort interaction between the apical hydrogen atom or its small substituent and the surface of the benzene ring. It is generally believed that this forced ultrashort X⋯π contact is associated with high steric hindrance, which is responsible for specific properties of iron maiden molecules. The main aim of this article is to investigate the influence of significant charge enrichment or depletion of the benzene ring on the characteristics of the ultrashort C-X⋯π contact in iron maiden molecules. For this purpose, three strongly electron-donating (-NH${}_{2}$) or strongly electron-withdrawing (-CN) groups were inserted into the benzene ring of *in*-[3${}^{4,10}$][7]metacyclophane and its halogenated (X = F, Cl, Br) derivatives. It is shown that, despite such extremely electron-donating or electron-accepting properties, the considered iron maiden molecules surprisingly reveal quite high resistance to changes in electronic properties.

## 1. Introduction

The so-called ‘iron maiden’ molecules [1,2,3,4,5,6,7,8,9] form one of the most interesting subgroups of *in*-cyclophanes [10,11]. They are characterized by the presence of a benzene ring with three bridges (usually aliphatic, but not necessarily) connected to each other in the keystone carbon atom that additionally forms a bond to a hydrogen atom (i.e., a C-H bond with methine H) or its substituent, e.g., a methyl group [6]. Crucially, this hydrogen atom is pointed towards the center of the ring. Such an arrangement of bonds (*in*) is often more stable than a structure in which the C-H bond is directed in the opposite direction to the ring (*out* arrangement) [5,12]. What is quite peculiar is that the compounds are named for the similarity of the final stage of ring closure during synthesis to medieval human-shaped torture devices, the so-called “iron maidens” in which spikes were driven into the victims enclosed in them [13]. Due to such a specific structure, iron maidens show interesting spectral properties, e.g., a fairly large blue-shift of the C-H stretching vibration frequency on the IR spectrum and a large ^1^H NMR resonance.

**Figure 1:**
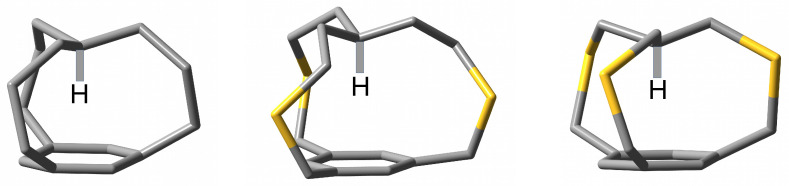
Structures of *in*-[3${}^{4,10}$][7]metacyclophane (**1**), 2,8,17-trithia-*in*-[4${}^{5,12}$][9]metacyclophane (**2**) and 2,6,15-trithia-*in*-[3${}^{4,10}$][7]metacyclophane (**3**) in sequence. All but the apical hydrogen atoms have been removed for clarity.

In the current work, the most important iron maiden compound is *in*-[3${}^{4,10}$][7]metacyclophane (**1**), which has three propenyl (i.e., -CH_2_-CH_2_-CH_2_-) bridges, as the other compounds studied can be considered as its derivatives. For the first time, this compound was synthesized in 1987 by Pascal Jr. et al. [2] by oxidation of the less spatially crowded sulfur derivative 2,8,17-trithia-*in*-[4${}^{5,12}$][9]metacyclophane (**2**) (having bridges -CH_2_-S-CH_2_-CH_2_-) by a mixture of 30% aqueous hydrogen peroxide and acetic acid followed by thermal extrusion of sulfur dioxide. Unfortunately, the authors failed to obtain a crystal and perform X-ray crystallographic analysis. Nevertheless, spectroscopic analysis showed a very large field resonance for the methine hydrogen atom (δ = −4.03 ppm) in the ^1^H NMR spectrum as well as a very large blue-shift of ca. 400 cm^−1^ for the $\nu_{\mathrm{CH}}$ frequency. This blue-shift was attributed to strong steric compression. Indeed, this is quite a telltale effect in steric constraint [14,15]. In addition to the spectroscopic measurements mentioned, Pascal et al. also performed molecular mechanics calculations (MM2(85)), which showed that the methine hydrogen is only 1.78 Å from the benzene ring [2]. It should be mentioned that, despite the failure to determine the X-ray crystallographic structure of *in*-[3${}^{4,10}$][7]metacyclophane (**1**), Pascal et al., however, managed to perform crystallographic analysis for its derivatives, e.g., 2,6,15-trithia-*in*-[3${}^{4,10}$][7]metacyclophane (**3**) [3] and 2,8,17-trithia-*in*-[4${}^{5,12}$][9]metacyclophane (**2**) [4]. The structural formulas of **1**–**3** are shown in Figure 1.

Figure 1 clearly shows that iron maiden molecules in the *in* conformation can be thought of as cage structures in which the methine hydrogen atom is tightly trapped between the keystone carbon atom, the ring plane and the aliphatic side chains. Very recently, we have studied the effect of replacing the methine hydrogen atom with a halogen atom X (X = F, Cl, Br) on the geometries and electronic structures of several iron maiden molecules with CCC, CCCC, CSC or CSCC side chains [9]. Many interesting findings have emerged. In terms of geometric changes, it has been shown that the H→F→Cl→Br substitution leads to a significant expansion of the cage structure of the iron maiden molecule. This structural expansion has been shown to be associated with an increase in the energetic destabilization of X. On the contrary, the H→F→Cl→Br substitution leads to an increasingly pronounced shortening of the C-X bond. Importantly, shorter side chains lead to stronger effects, while insertion of sulfur atoms into the side chains reduces these effects relative to the carbon counterpart [9].

Interestingly, based on the obtained MBO (Mayer Bond Order [16,17,18,19], which is a generalization of Wiberg Bond Index [20]) values, it has been shown that the Br⋯π interaction is bonding regardless of the molecule, the Cl⋯π interaction is bonding in molecules with longer side chains (i.e., CCCC or CSCC) and anti-bonding when the side chains are short (i.e., CCC or CSC), while the interaction F⋯π is to be anti-bonding regardless of the type of molecule. Nevertheless, regardless of whether the MBO values suggest a bonding or anti-bonding nature of the X⋯π interaction, the QTAIM-based (Quantum Theory of Atoms in Molecules [21,22,23,24,25]) calculations have always given three bond paths between the apical X atom and the carbon atoms of the benzene ring. Therefore, if the X⋯π interaction were to be anti-bonding indeed, the presence of such bond paths should be considered counterintuitive [26,27,28,29,30,31] and contrary to the orthodox QTAIM [24,25]. On the other hand, the QTAIM-based IQA (Interaction Quantum Atoms [32,33]) energy decomposition scheme has shown that the X⋯π interaction in each of the analyzed iron maiden molecules is stabilizing and, interestingly, almost purely covalent in nature, as indicated by the overwhelming dominance (>95%) of the exchange-correlation energy term [9]. To this picture of the X-substituted iron maidens must be added a rather complex pattern of the repulsive and attractive interactions inside the cage structure as has been obtained using NCI (i.e., the Noncovalent Interactions index [34,35]). Namely, the X⋯π interaction has turned out to be composed of areas corresponding to both of these contributions (i.e., the repulsive and attractive interactions) and not just one of them. Most importantly, however, it has turned out that the substitution H→F→Cl→Br leads to the expansion of a repulsive region between the apical X atom and the side chains, especially the C-C bonds. Following this finding, we have proposed [9] that the steric hindrance in the *in* conformers of iron maiden molecules is not so much due to the repulsion between the apical X atom and the benzene ring, but rather to unfavorable steric interactions between the X and the C-C side bonds.

At the time of our earlier article on iron maiden molecules [9], we were unaware of two slightly earlier theoretical papers on the subject [7,8]. In the first of them, Vujović et al. [7] have applied ETS-NOCV (i.e., the combination of the Extended Transition State (ETS) method [36] with the Natural Orbitals for Chemical Valence (NOCV) method [37,38,39]) and DF-SAPT (Density-Fitting Symmetry-Adapted Perturbation Theory [40,41]) interaction energy decomposition methods to describe *in*/*out* isomerism in **1** and **3** as well as their X-substituted derivatives. Most importantly, Vujović et al. [7] have shown that the greater stability of *in* forms of **1** and **3** results mainly from orbital stabilization with the participation of the aromatic ring electron density. However, the result may depend on the fragmentation scheme of the molecule within ETS-NOCV, in this case to the methine hydrogen or halogen and the rest of the molecule. In the case of SAPT-based calculations, the dimer model [42] was used, in which the interaction in cyclophanes was modeled using the dimer 1,3,5-trimethylbenzene⋯methane. Interactions between fragments of such a dimer have turned out to be repulsive, thus suggesting the repulsive nature of the H/X⋯π contact in the considered *in* forms of the iron maiden molecules [7]. However, it should be noted at this point that both ETS-NOCV and SAPT give results dependent on the interaction between whole fragments, while IQA may give a more local insight [9]. In the second article, Østrøm et al. [8], in addition to molecules **1** and **3**, also examined a series of their derivatives in which the central atoms of the side chains were replaced by NH${}_{2}^{+}$, NH or O and the benzene (or 1,3,5-triazine) ring contained three substituents. It turned out that, among the considered systems, the shortest C-H⋯π contact occurred in the derivative of **3** containing a 1,3,5-triazine ring and oxygen atoms built in place of sulfur atoms. Nevertheless, the *out* conformer was more energetically favorable in this case.

**Figure 2:**
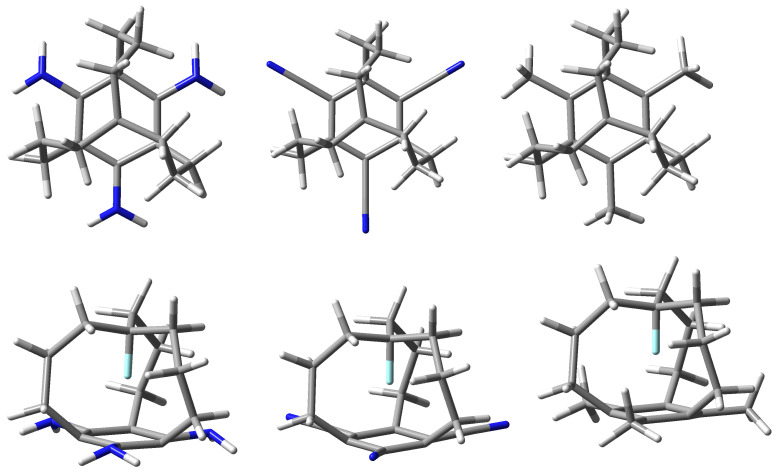
Top and side views of the structures of -NH${}_{2}$ (left), -CN (center) or -CH${}_{3}$ (right)-substituted X-derivatives of *in*-[3${}^{4,10}$][7]metacyclophane (**1**). Individual types of atoms are marked with the following colors: carbon—black, hydrogen—white, nitrogen—blue, X—cyan.

To shed more light on the structural relationships and electronic structure of molecular iron maidens, derivatives of *in*-[3${}^{4,10}$][7]metacyclophane (i.e., **1**) and its X-substituted (X = F, Cl, Br) counterparts in which, additionally, hydrogen atoms from the benzene ring have been replaced with amino, nitrile or methyl groups (Figure 2) are studied in this work.

As the parent molecule, **1** has been chosen because, as mentioned earlier, having short CCC carbon chains, it has been characterized by the most pronounced effects [9]. On the other hand, the amino group is one of the most electron-donating substituent, while on the contrary, the nitrile group is one of the most electron-withdrawing [43,44,45]. In addition, the number of as many as three amino or nitrile groups provides the greatest electronic effect and thus the greatest impact on the distribution of electron density in the benzene ring. In this way, the entire range of electronic properties of substituents is covered because other substituents or a different number of them should only lead to intermediate effects. Nevertheless, derivatives with three methyl groups have also been considered to check the influence of the presence of substituents creating only (almost) pure inductive effect. It should be noted that the number of three substituents preserves the symmetry of the molecule (C${}_{3}$ symmetry point group). Otherwise, this symmetry would be broken, which would significantly complicate the presentation of the obtained results. The main purpose of this paper is to present the effect of charge enrichment or depletion of the benzene ring on the structure of X-derivatives of molecule **1** and on the characteristics of the X⋯π ultrashort contacts. The presented results also perfectly complement the findings of earlier theoretical works [7,8] on molecular iron maidens.

## 2. Results and Discussion

### 2.1. Structure of Iron Maidens

As already described in the Introduction and shown in Figure 1 and Figure 2, iron maiden molecules are characterized by a cage structure formed by a benzene ring (or another obtained by its modification) from which three aliphatic (most often) bridges are combined in the keystone carbon atom forming an additional C-H bond directed in conformer *in* towards the center of the ring. This orientation leads to an ultrashort contact H⋯π. For consistency, we will start a thorough analysis of the structure of iron maiden molecules by presenting the influence of the H→F→Cl→ Br substitution. Next, the influence of the presence of three -NH${}_{2}$, -CN or -CH${}_{3}$ groups in the benzene ring will be discussed.

#### 2.1.1. Influence of the H→F→Cl→Br Substitution

**Figure 3:**
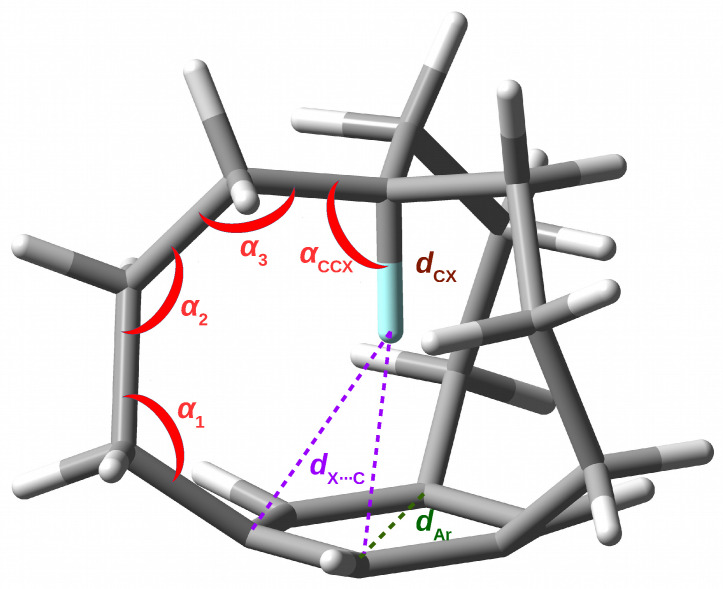
Meaning of some structural parameters present in Table 1.

The values of the most important structural parameters characterizing the investigated *in* forms of the **Xn** iron maiden molecules are shown in Table 1. The meaning of these parameters is shown in Figure 3 and explained in the footnotes to Table 1.

The data clearly show an increase in the size of the **X0** molecule during the H→F→Cl→Br substitution. This effect is manifested by a monotonic increase in the distances X⋯π (or X⋯RCP), C⋯π, X⋯C as well as the size of the benzene ring and the angles $\alpha_{1}$ and $\alpha_{2}$. However, in the case of $\alpha_{3}$, only the H→Br substitution gives an increase in the value, while H→Cl and especially H→F give a decrease. The expansion of the molecular size leads to a greater flattening of the fragment containing the keystone carbon atom, which is visible in the ever greater deviation of the $\alpha_{\mathrm{CCX}}$ angle (104${}^{\circ }$→ 99${}^{\circ }$→ 95${}^{\circ }$→ 93${}^{\circ }$) from the 109.5${}^{\circ }$ value characteristic for the sp${}^{3}$ hybridization. The X-substituted **H0** derivatives are characterized by greater folding of the benzene ring.

As for the C-X⋯π contact, as already mentioned, the H→F→Cl →Br substitution leads to a rapid increase in the X⋯ring distance. Due to the ring corrugation, in addition to the distance between X and the centroid of the ring ($d_{X\cdots \pi }$), the distance to the RCP of this ring ($d_{X\cdots \mathrm{RCP}}$) has also been determined. Of course, the relation $d_{X\cdots \pi }>d_{X\cdots \mathrm{RCP}}$ is visible because the RCP is slightly above the benzene ring. Of particular importance is the analysis of the C-X bond length. Although the value of its change depends on the reference molecule, the data presented in Table 1 clearly indicate a significant shortening of this bond, especially in relation to the *out* form (−0.033, −0.103, −0.234 and −0.282 Å for H, F, Cl and Br, respectively). By far, the largest conspicuous shortenings for Cl and Br are due to the extremely long C-Cl and C-Br bonds in the *out* forms. For example, in the case of **Br0**, the C-Br bond shortens from 2.056 Å in the *out* form to only 1.774 Å in the *in* form. The shortening of the C-X bond should lead to an increase in the stretching vibration frequency (blue-shift) [14,15]. It is worth recalling here that, for the **H0** molecule, Pascal et al. have measured a blue-shift of as much as 400 cm${}^{-1}$ [2].

#### 2.1.2. Influence of Substituents in the Benzene Ring

Leaving behind the brief discussion of the effect of the H→F→Cl→ Br substitution on the structure of iron maiden molecules, we now move on to the discussion of the influence of the presence of a substituent on the benzene ring. As already underlined in the Introduction, in order to consider the largest possible range of electronic effects, the hydrogen atoms in the benzene ring of the parent **X0** molecules have been replaced with as many as three -NH${}_{2}$ or -CN groups (see Figure 2). The former is characterized by one of the largest electron-donating properties, while the latter one by one of the largest electron-withdrawing effects. Thus, other possible substituents should only give an intermediate effect [43,44,45]. As a consequence, the presence of -NH${}_{2}$ groups should significantly increase the electron density within the ring (actually, in positions *para* and *ortho* to this group [43,44,45]), while the presence of -CN groups should significantly decrease it. Unlike both of these groups, the -CH${}_{3}$ group influences the distribution of the electron density in the benzene ring (almost) only through the inductive effect. One of the possibilities to verify the magnitude of the electron charge in the ring is to determine the value of the electron density in the RCP of this ring. These values are given in Table 2.

Unfortunately, they suggest that -NH${}_{2}$, -CN or -CH${}_{3}$ substitution leads to a decrease of $\rho_{\mathrm{RCP}}$. However, the changes are so tiny that they can hardly be considered a compelling indicator. Therefore, the electron density distribution in the ring has also been probed by means of atomic charges. In order to determine them, we decided to use the Hirshfeld method [46] as Hirshfeld atomic charges have been shown to be chemically reliable [47,48]. It should be recalled here that there are two types of carbon atoms present in the ring: those that are attached to the side chains and those that are not (Figure 2). The charges of both are also shown in Table 2. In addition, the total charge on all carbon atoms of the benzene ring has also been determined ($Q_{C}^{\mathrm{ring}}$ in Table 2). Let us first note that the presence of a halogen atom in an unsubstituted **X0** iron maiden molecule is associated with a greater value of negative charge (ca. −0.22 vs. −0.14 a.u. in **H0**). The insertion of three -CN groups does indeed conspicuously draw the electron charge from the ring, so that $Q_{C}^{\mathrm{ring}}$ becomes distinctly positive regardless of the type of the X atom. The change in $Q_{C}^{\mathrm{ring}}$ is almost constant and amounts to ca. 0.3 a.u. In the case of the -NH${}_{2}$ group, the situation revealed by the atomic charges is more complex. As this group crowds electrons into the *para* and *ortho* positions, a significant increase in the negative atomic charge on the chain carbon atoms of the ring is to be expected. Table 2 shows that this is indeed the case. Interestingly, however, the results show that this gain of electron charge on the chained ring carbon atoms is overcompensated by the loss on the non-chained carbon atoms, i.e., those directly bonded to -NH${}_{2}$ groups. This is the result of the weak −I (inductive) effect of the amino group. As a result of the suggested predominance of the latter effect, $Q_{C}^{\mathrm{ring}}$ becomes slightly less negative. However, another distribution of atomic charges in the ring is present in the case of compounds with three -CH${}_{3}$ groups. Namely, as in their unsubstituted counterparts, the ring carbon atoms are endowed with partial negative charges, but it is worth noting that these charges are *smaller* (i.e., less negative) than in **X0**. Such a result means that the presence of methyl groups *draws* the electron charge from the benzene ring, which is contrary to their generally accepted +*I* nature. On the other hand, this result is consistent with a negative sEDA (sigma-Electron-Donor-Acceptor) value [49], suggesting −*I* rather than a +*I* effect. Of course, the charge is drawn from the *ipso*-carbon atoms and its amount (ca. 0.05 a.u.) does not depend on the type of X atom. On the other hand, a smaller amount of charge (ca. 0.02 a.u.) is pumped onto the *ortho* carbons, so that the presence of three methyl groups depletes the benzene ring by a constant value of approx. 0.1 a.u.

More important, however, is the analysis of the influence of -NH${}_{2}$, -CN or -CH${}_{3}$ substituents on the structure of the iron maiden compounds under consideration. As can be seen from the results presented in Table 1, the insertion of three -CN groups into the benzene ring significantly increases its folding and size. However, although the former effect increases steadily in the H (0.012 Å) → F (0.028 Å) → Cl (0.045 Å) → Br (0.058 Å) order, the latter effect is practically constant (0.015 Å). Similarly, regardless of X, a small and almost constant reduction (by 0.5${}^{\circ }$) of the $\alpha_{1}$ angle has been obtained. On the other hand, the angle $\alpha_{3}$ increases slightly as the radius of the atom X increases: H (0.2${}^{\circ }$) < F (0.4${}^{\circ }$) < Cl (0.5${}^{\circ }$) < Br (0.6${}^{\circ }$). On the contrary, the influence on $\alpha_{\mathrm{CCX}}$ is the opposite and negligible: H (−0.4${}^{\circ }$) > F (−0.2${}^{\circ }$) > Cl (−0.1${}^{\circ }$) > Br (0.0${}^{\circ }$). The greatest but also small changes concern the $\alpha_{2}$ angle: **H2** (+0.9${}^{\circ }$) and **Br2** (−0.7${}^{\circ }$). Regarding the X⋯π distance, the greatest change (shortening) has been obtained when X = F (−0.025 Å) and much smaller when X = Cl (−0.005 Å). Interestingly, the presence of three -CN groups in the benzene ring has no effect on the X⋯π distance when X = H (0.000 Å) or Br (+0.002 Å). Apart from perhaps **F2** (−0.010 Å), the influence of the presence of -CN groups on the C-X bond length is also rather small: **H2** (−0.002 Å), **Cl2** (+0.001 Å), **Br2** (−0.004 Å). This is quite a surprising result, as it shows a fairly high resistance of the iron maiden molecules to the presence of symmetrically arranged substituents strongly draining the electron charge from the benzene ring.

In the case of the presence of three -NH${}_{2}$ groups, practically only one agreement with the identical effect induced by the presence of three -CN groups has been obtained. Namely, the presence of these groups also leads to an increase in the size of the benzene ring. However, unlike the -CN groups, where this increase (0.015 Å) was independent of the type of X atom, in the case of -NH${}_{2}$, the size of the effect increases with the size of the X atom: H (+0.021 Å) < F (0.025 Å) < Cl (0.034 Å) < Br (0.041 Å). In contrast to the previously discussed changes caused by the presence of -CN groups, the presence of -NH${}_{2}$ groups reduces rather than increases the folding of the benzene ring: H (−0.011 Å) < F (−0.035 Å) < Cl (−0.043 Å) > Br (−0.038 Å). As for the values of the angles, the changes are also opposite. The presence of three -NH${}_{2}$ groups leads to an increase (and not a decrease) in the value of the $\alpha_{1}$ angle (by about 1.4${}^{\circ }$), a slight decrease (and not an increase) in the value of the $\alpha_{3}$ angle (−0.2${}^{\circ }$), and a systematic slight increase (and not a decrease) in the value of the $\alpha_{\mathrm{CCX}}$ angle: H (+0.2${}^{\circ }$) < F (+0.3${}^{\circ }$) < Cl (+0.6${}^{\circ }$) < Br (+0.7${}^{\circ }$). In addition, the value of the angle $\alpha_{2}$ is lowered (up to −0.9${}^{\circ }$ for X = halogen). Most interestingly, the presence of three -NH${}_{2}$ groups in the benzene ring independently of X leads to elongation of the C-X bond: H/F (+0.003 Å) < Cl (+0.007 Å) < Br (+0.008 Å). As with the three -CN groups, the influence of the three -NH${}_{2}$ groups on the X⋯π distance is more complex. Namely, in the case of **H1** and **F1**, longer distances have been obtained (by +0.012 and +0.005 Å, respectively), while in the case of **Cl1** and **Br1**, shorter ones (by −0.012 and −0.022 Å, respectively). It is worth noting that, despite the smaller folding of the benzene ring, the presence of -NH${}_{2}$ groups increases the distance between the centroid of the ring and its RCP. This increase is the largest in the case of X = Br and amounts to 0.026 Å.

The presence of three methyl groups in the benzene ring only slightly affects the length of the C-X bond; in the case of X = F, it is shorter by −0.001 Å, while, in the case of X ≠ F, it is slightly longer, by +0.003 Å in the case of Cl and Br. On the contrary, the distance X⋯π (and C⋯π) decreases regardless of the type of atom X. For the former distance, this shortening (−0.005 Å) does not depend on X and only for Br is it greater (−0.010 Å). As in the case of -CN, the presence of three -CH${}_{3}$ groups causes an increase in the folding of the benzene ring; however, unlike in the case of -CN, this increase does not intensify with the size of the X atom, and on the contrary, it is the largest (+0.016) when X = H (however, for the remaining X, the range of corrugation is clearly larger). Again, as with the -CN groups, the presence of three -CH${}_{3}$ groups in the benzene ring increases the ring size, by a constant value of 0.017 Å. Unlike -CN and -NH${}_{2}$, the presence of -CH${}_{3}$ groups increases the values of all considered angles, with the increase being the greatest in the case of $\alpha_{2}$ and ranging from +0.7${}^{\circ }$ for **H3** to +1.3${}^{\circ }$ for **Cl3**. Changes in these angles do not depend on the radius of the X atom, but in the case of $\alpha_{3}$ the increment is constant at +0.3${}^{\circ }$. The influence of the three -NH${}_{2}$, -CN or -CH${}_{3}$ groups on the structural parameters of the iron maiden molecules under consideration is summarized in Table 3. It is worth noting that, in terms of the influence on the values of the considered geometric parameters, the -CH${}_{3}$ group behaves much more similarly to the -CN group than to -NH${}_{2}$.

### 2.2. Energetic Stability of *in* Forms

#### 2.2.1. *Out* → *In* Isomerization Energy

We have recently shown [9] that, in the case of **X0** and their derivatives with CCCC, CSC or CSCC side chains, the *out* →*in* isomerization is exothermic only when X = H, while in the case of X = halogen, the process is endothermic and larger with increasing the radius of X. The *out*→ *in* isomerization energies obtained for **X1**, **X2** and **X3** are listed in Table 4. The values obtained earlier for **X0** are also shown for comparison.

In the case of X = H, the presence of either -NH${}_{2}$ or -CH${}_{3}$ groups in the benzene ring stabilizes the *in* conformer to a slightly greater extent (by 0.5 and 0.8 kcal/mol, respectively) than the reference *out* conformer of **H0**. The -CN groups have the opposite effect, i.e., they reduce this relative stabilization (by 1.7 kcal/mol). This direction of changes supports the suggestion that the H⋯π interaction in **Hn** molecules is stabilizing because enrichment of the benzene ring in electron charge should strengthen this interaction, while, conversely, depletion of the ring should weaken this interaction. Interestingly, the presence of three groups -NH${}_{2}$, -CN, or -CH${}_{3}$ reduces the endothermic effect of the *out*→*in* isomerization process when the apical H atom is substituted by a halogen (the only exception is **F3** with a slight increase in isomerization energy of +0.9 kcal/mol). However, with -NH${}_{2}$, this effect increases in the F (−2.8 kcal/mol) < Cl (−10.3 kcal/mol) < Br (−13.8 kcal/mol) order, while with -CN, the order is reversed: Br (−1.8 kcal/mol) < Cl (−3.8 kcal/mol) < F (−6.2 kcal/mol). Assuming that the interactions H⋯π, Cl⋯π and Br⋯π are attractive while the interaction F⋯π is repulsive, the results shown in Table 4 suggest that the -CH${}_{3}$ group creates a +*I* effect rather than −*I*. On the other hand, however, changes in *out*→*in* isomerization energies may result mainly from changes in bond stresses and not only from the interaction strength of X⋯π.

#### 2.2.2. Energy of the X Substituent

A very valuable parameter that can be used to describe the energetic stabilization/destabilization of the substituent X is its energy [50] obtained (Equation (3)) according to the homodesmotic reaction 4. The substituent energy, $\Delta E_{\mathrm{Xn}}^{in/out}$(X), well describes proximity effects [50] and therefore is also great for describing the effect of the cage environment on the apical X atom in iron maiden molecules [9]. The obtained substituent energy values are shown in Table 5.

**Figure 4:**
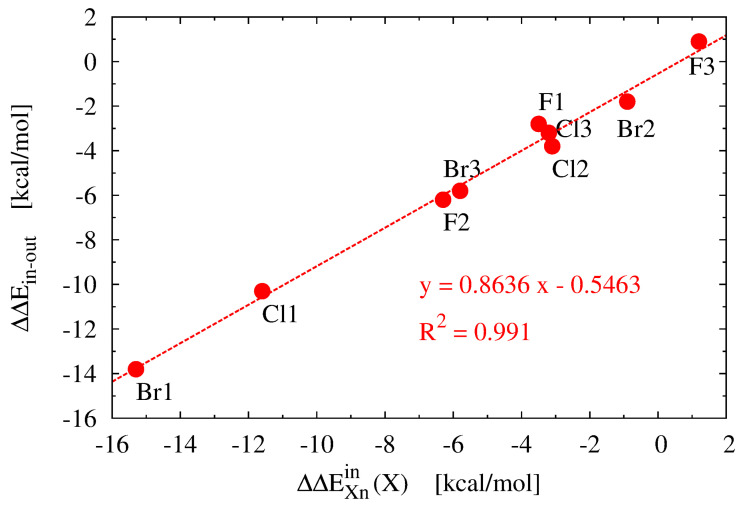
The relationship between the change in the *out* →*in* isomerization energy caused by the substitution of three groups -NH${}_{2}$, -CN or -CH${}_{3}$ to the benzene ring of the iron maiden **X0** molecules and the change in the energy of the X (X = F, Cl, Br) substituent (see Table 4 and Table 5).

It is clear that the obtained $\Delta E_{\mathrm{Xn}}^{in}$ values for the *in* forms are positive, showing that the X substituents (i.e., the halogen atoms) are less energetically stable than in the reference CH_3_X molecules. Regardless of **n**, the X-substituent energy value increases rapidly with the size of the X atom, i.e., F < Cl < Br. For **X0**, the corresponding values are as follows: 48.6, 179.2 and 240.6 kcal/mol. Interestingly, the presence of three -NH_2_, -CN, or -CH_3_ groups leads to a slightly greater stabilization of X in these forms, so that the destabilization value decreases somewhat (the only exception here is of course **F3** showing an increase in the destabilization of the F atom by +1.2 kcal/mol). In the case of -NH_2_ (**X1**), the influence of substitution increases in the F (−3.5 kcal/mol) < Cl (−11.6 kcal/mol) < Br (−15.3 kcal/mol) direction, while in the case of substitution of -CN groups (**X2**), the order is reversed: Br (−0.9 kcal/mol) < Cl (−3.1 kcal/mol) < F (−6.3 kcal/mol). Importantly, these sequences are the same as the previously discussed sequences of the effect of substitution of -NH_2_ or -CN groups on the *out*→*in* isomerization energy (see Table 4). This obtained correlation suggests that the X substituent has a strong influence on the *out*→*in* isomerization energy. This is confirmed by the excellent linear correlation (R^2^ = 0.991) between the two effects (see Figure 4). The reduction in X destabilization after the introduction of -NH_2_, -CN or -CH_3_ groups may be due to the increase in ring size in both cases (Table 3). By comparing the obtained energies of the X substituents in the *in* forms of **Xn** to the energy of the -NO_2_ group in the sterically most crowded ’bay’ position 4 in phenanthrene, which was only +10.0 kcal/mol (B3LYP/6-311++G(2df,2p)) [50], we realize that the apical X substituent suffers from a really considerable steric hindrance inside the iron maiden molecule.

**Figure 5:**
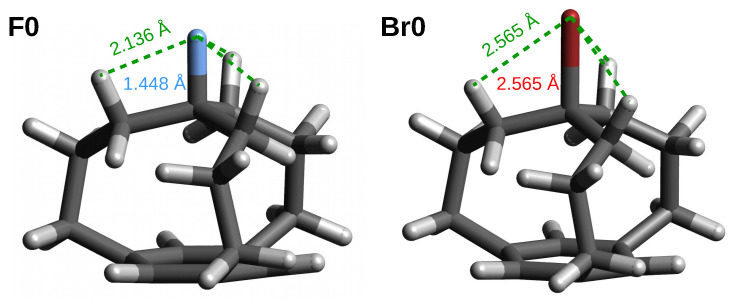
The *out* forms of the **F0** and **Br0** iron maiden molecules.

In contrast to the *in* forms, the X substituent is more stable in the *out* forms than in the reference molecule CH_3_X, as indicated by negative (except **Br2**) energies of the X substituents (Table 5). Apart from **Fn**, however, these energies are rather small; the stabilization energy of X does not exceed −6.0 kcal/mol. Clearly, greater stabilization of the F atom in the *out* forms of **Fn** compared to CH_3_F (from −12.5 to −15.4 kcal/mol) is probably due to the fact that, in the *out* forms, the F atom appears vaguely in a ’basket’ formed by three C-H bonds almost parallel to the C-F bond, which allows for three F⋯H contacts. This is shown for a representative **F0** molecule along with a comparison to the situation in **Br0** in Figure 5. In the case of **F0**, the F⋯H distances are only 2.136 Å while, in the case of **Br0**, the Br⋯H distances are as long as 2.565 Å, which is not conducive to the stabilization of the Br atom.

In the case of the *out* conformers, a difference in the effect on the X substituent energy has been obtained depending on whether -NH_2_ or -CN groups were inserted into the benzene ring. Namely, the presence of three amino groups increases the stabilization of the X atom, while the presence of three nitrile groups decreases it. In both cases, the effect is rather small (from −1.9 to 2.6 kcal/mol). In this respect, the -CH_3_ group behaves similarly to -NH_2_; however, the increase in stabilization of the X atom is lower by about 1 kcal/mol.

### 2.3. Strength of X⋯π According to WBI and MBO

The strength of bonds, especially in organic chemistry, is eagerly described using the popular Wiberg Bond Index (WBI) [20]. Considering the two types of carbon atoms in the benzene ring of **Xn**, WBIs were determined for both the X⋯C${}_{\mathrm{ring}}^{\mathrm{chain}}$ and X⋯C${}_{\mathrm{ring}}^{\mathrm{no}-\mathrm{chain}}$ interactions. The corresponding values, together with the total value obtained by summing over all X⋯C${}_{\mathrm{ring}}^{\mathrm{chain}/\mathrm{no}-\mathrm{chain}}$ interactions, are listed in Table 6.

The WBI values obtained for X⋯C${}_{\mathrm{ring}}^{\mathrm{chain}}$ are slightly greater than for X⋯C${}_{\mathrm{ring}}^{\mathrm{no}-\mathrm{chain}}$, which results from the shorter distances of X⋯C${}_{\mathrm{ring}}^{\mathrm{chain}}$ compared to X⋯C${}_{\mathrm{ring}}^{\mathrm{no}-\mathrm{chain}}$ (Table 1). However, the differences are negligible. Most importantly, the obtained data indicate an increase in the WBI value for the total contact X⋯π with the increase of the radius of the X atom, and thus suggest an increase in the strength of this interaction in this direction. Interestingly, the results indicate an increase in the strength of X⋯π interaction regardless of the type of substituent in the benzene ring, but the greatest effect is visible in the case of -NH${}_{2}$, while the smallest in the case of -CH${}_{3}$.

Unlike MBO, WBI always gives positive values, which follows directly from the definition of this bond order index (see Equation (5)). Therefore, this index is unable to show the anti-bonding nature of a bond. For this reason, it is particularly interesting to compare the obtained WBI values with their MBO counterparts, which are also included in Table 6. The values in the last column of Table 6 indicate a very high sensitivity to the type of substituents in the benzene ring (and of course to the type of X atom). This is partly due to the high dependence of the MBO value on the type of carbon atom in the ring, so it should be taken into account during the analysis. In the case of the presence of -NH${}_{2}$ groups, one should take into account the fact that the distribution of electron density within the benzene ring is definitely differentiated with enriched *para* and *ortho* (i.e., chain) carbons and depletion of the charge on the C atoms to which the -NH${}_{2}$ groups are attached (Table 2). If the bonding effect (0.083) obtained for **Br0** is to be explained by the presence of a well-marked σ-hole [51,52] on the Br atom, and the clear anti-bonding effect (−0.342) for **F0** is to be explained by the repulsion of the strongly electronegative F atom with ring π-electrons [9], then the enrichment of carbon atoms in the ring with an electron charge should increase the bonding (anti-bonding) effect of X⋯C${}_{\mathrm{ring}}^{\mathrm{chain}}$ in **Br1** and **F1**, respectively, which is indeed the case (0.372 and −0.118, respectively). The direction of changes (for X⋯C${}_{\mathrm{ring}}^{\mathrm{chain}}$) in the MBO values obtained for **Cl1** and **H1** is also easily explained. Namely, in the case of **Cl1**, the increase in the bonding effect (from 0.032 to 0.124) can be explained by the stronger σ-hole⋯C${}_{\mathrm{ring}}^{\mathrm{chain}}$ interaction, while the decrease in the anti-bonding effect at the **H0**→**H1** transition can be explained by the greater binding contribution in the H⋯C${}_{\mathrm{ring}}^{\mathrm{chain}}$ interaction. However, the bonding/anti-bonding effects of the X⋯C${}_{\mathrm{ring}}^{\mathrm{chain}}$ interactions compete with those from X⋯C${}_{\mathrm{ring}}^{\mathrm{no}-\mathrm{chain}}$. Charge depletion of the no-chain C atoms (Table 2) leads to a shift towards an anti-bonding effect, with the exception of **F1**, for which the weaker F⋯C repulsion leads to bonding X⋯C${}_{\mathrm{ring}}^{\mathrm{no}-\mathrm{chain}}$ contacts (from −0.011 in **F0** to 0.073 in **F1**). As a consequence, the anti-bonding character of the X⋯C${}_{\mathrm{ring}}^{\mathrm{no}-\mathrm{chain}}$ interactions in **Cl1** and **Br1** strengthens (to ca. −0.42). The nature of the X⋯π interaction depends on the nature of the X⋯C${}_{\mathrm{ring}}^{\mathrm{chain}}$ and X⋯C${}_{\mathrm{ring}}^{\mathrm{no}-\mathrm{chain}}$ interactions. The MBO values for X⋯π presented in Table 6 suggest that each of the X⋯π interactions within the **X1** molecules is anti-bonding, regardless of the type of X atom.

On the other hand, the insertion of three -CN groups into the benzene ring leads to a very significant removal of the electron charge (see Table 2 ). This should weaken the σ-hole⋯π interaction in the case of X = Cl and Br, which is indeed observed, since the X⋯π interactions are suggested to be anti-bonding according to the MBO (−2.415 and −0.494, respectively). However, such a large difference in the obtained values seems to be somewhat suspicious. In the case of X = F, the smaller anti-bonding effect (−0.112 in **F2** vs. −0.342 in **F0**) can be explained by the weaker F⋯π repulsion resulting from the charge flow from the ring. According to the MBO values, the anti-bonding nature of the H⋯π interaction in **H2** is to be similarly strong as in **H0** (−0.190 and −0.136, respectively).

It is worth commenting on the positive MBO value (0.095) obtained for **Br3**, i.e., the species with a bromine atom as X and three -CH${}_{3}$ groups in the benzene ring. As this value is similar to the value obtained for the unsubstituted counterpart **Br0** (0.083), it simply suggests a small influence of three -CH${}_{3}$ groups. However, this value is due to opposing changes on the chain and no-chain carbon atoms of the benzene ring. Namely, the attachment of three -CH${}_{3}$ groups leads to a slight inflow of electron charge on the chain-carbon atoms (Table 2) and thus to an increase in the Br⋯C${}_{\mathrm{ring}}^{\mathrm{chain}}$ bonding effect (from 0.100 in **Br0** to 0.198 in **Br3**; Table 6). Conversely, the outflow of charge from the no-chain carbons increases the anti-bonding effect (from −0.072 in **Br0** to −0.166 in **Br3**). Since the former of these effects is slightly larger, an increase in the bonding effect is finally obtained. It is worth noting that, although the increase in bonding effect could simply be explained by the alleged +*I* inductive effect of the methyl group, such a simplistic approach would overlook the noted opposite effects on both types of ring carbon atoms.

### 2.4. QTAIM-Based Analysis

The influence of the presence of three -NH${}_{2}$, -CN or -CH${}_{3}$ substituents on the characteristics of the iron maiden molecules in question can be further investigated using QTAIM [21,22,23,24,25]. Within this theory, the interaction between the apical X atom and the benzene ring can be successfully described by a series of parameters determined at the BCPs of the bond paths tracing the X⋯C${}_{\mathrm{ring}}$ contacts. The QTAIM calculations have always yielded only three bond paths of this type. The most important QTAIM-based parameters determined in the BCP of this interaction are listed in Table 7.

It is quite surprising that the values of the analyzed parameters (perhaps with the exception of λ and ε) do not change significantly after inserting as many as three -NH${}_{2}$, -CN or -CH${}_{3}$ groups into the benzene ring. This shows quite a high resistance of iron maiden molecules to changes in electronic properties. Although the -NH${}_{2}$ and -CN groups have sufficiently strong either electron-donor (+*M*) or electron-acceptor (−*M*) properties, respectively, for a given X, the changes in $\rho_{X\cdots C}$ are practically negligible. For the more sensitive Laplacian, although the changes are more noticeable, they are also small. In the case of $\rho_{X\cdots C}$, the values increase in the order H < F < Cl ≤ Br suggesting an increase in the X⋯π interaction strength in this order. $H_{X\cdots C}$ values are close to zero; however, in the case of Cl and Br, they are negative, indicating a small covalent contribution. The values depend negligibly on the type of substituent on the benzene ring.

In our previous studies of iron maiden molecules, we have reported an unusually high value of the X⋯C bond ellipticity (32) found for the compound with an apical fluorine atom and three four-carbon side chains [9]. This result has been attributed to the extremely flat electron density distribution in the $\lambda_{2}$ eigenvalue plane (see Figure 6 in [9]). Although in the case of the **Xn** iron maiden molecules studied here, the values of the X⋯C bond ellipticity are much lower (ca. 2–16), they are clearly higher than, for example, in the case of agostic bonds [53,54,55,56,57,58], for which it is commonly said that the bond ellipticity values are significant [59,60,61], although they are generally lower than 1–2 [62,63,64]. Moreover, in **H0** and especially in **H2**, these values are also notably large (11 and 16, respectively). Since the bond ellipticity ε is defined as $\lambda_{1}$/$\lambda_{2}-1$, its large value must be due to the much smaller value of $\lambda_{2}$ relative to $\lambda_{1}$. The results shown in Table 7 indicate that this is indeed the case, especially for **H2**, of course (−0.0013 and −0.0229, respectively). It is also worth noting that, in this case, the absolute value of $\lambda_{2}$ is almost equal to the positive value of $\lambda_{2}^{\mathrm{RCP}}$ (0.0011), determined at the RCP lying in the same plane as the BCP of the H⋯C interaction. This fact confirms the considerable flatness of the electron density distribution in the $\lambda_{2}$ direction.

Although, as already mentioned in the Introduction section, the QTAIM calculations have always yielded three X⋯C${}_{\mathrm{ring}}$ bond paths, this does not mean that the apical X atom interacts only with three carbon atoms. It must be remembered that the presence of a bond path (BP) in general has nothing to do with the energy of the interaction traced by this bond path [26,27,28,29,30,31] since BP may not exist for a stabilizing interaction [27,28,65] and, conversely, it may exist even for a non-stabilizing, i.e., repulsive, interaction [26,27,28,29,30,31,66,67,68,69,70,71]. Therefore, in the case of the delocalization index, it is more reasonable to consider its total value, obtained by summing up over all individual contributions (see Equation (7)). Together with its equivalent taking into account distances [72] X⋯C (see Equation (8)), its values determined for the **Xn** iron maiden molecules are shown in the last two columns of Table 7. Similar to the previously discussed $\rho_{X\cdots C}$, $\nabla^{2}\rho_{X\cdots C}$ and $H_{X\cdots C}$ computed at BCP on the X⋯C bond path, the effect of substituting three groups -NH${}_{2}$, -CN or -CH${}_{3}$ on the benzene ring is small, although in some cases clear. The greatest $\delta^{\mathrm{tot}}$(X,π) changes have been obtained for **F1** (−0.013), **F2** (0.009), **Br1** (0.014) and **Br2** (−0.012). Due to the small influence of the functional groups substituted on the ring, the value of $\delta^{\mathrm{tot}}$(X,π) depends mainly on the type of atom X and increases with the increase of its radius: H (ca. 0.21) < F (0.36–0.39), < Cl (0.62) < Br (ca. 0.70–0.73). These values can be compared with ca. 0.16–0.40 obtained by Foroutan-Nejad et al. [73] for X${}^{-}\cdots \pi$ interactions and ca. 0.14–0.18 obtained by Badri et al. [74] for O⋯π contacts in various water ⋯π-ring systems. Evidently, the values for the considered iron maiden molecules are much larger, thus showing that the X⋯π interactions are relatively strong and multi-center. Change trends in $\delta_{\mathrm{RP}}^{\mathrm{tot}}$(X,π) follow trends in changes in $\delta^{\mathrm{tot}}$(X,π), but both the values themselves and these changes are obviously smaller.

**Figure 6:**
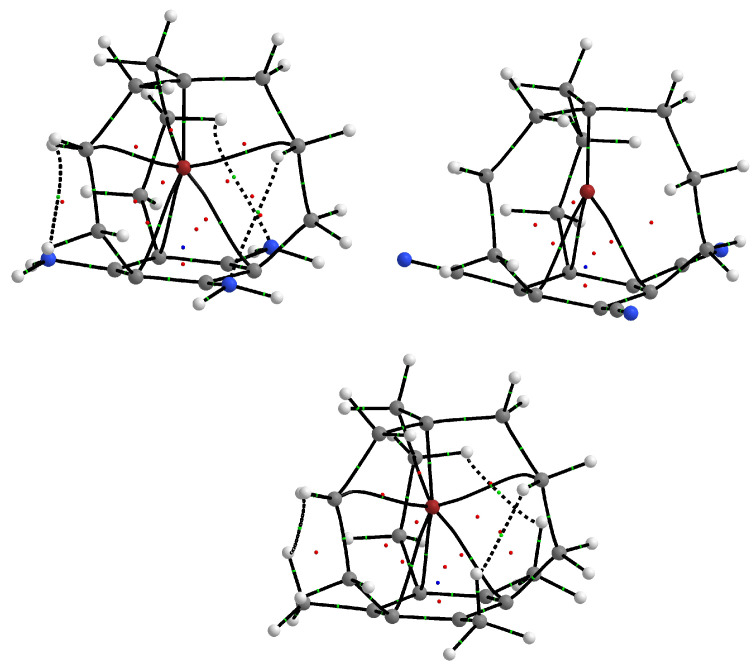
Molecular graphs of **Br1**, **Br2** and **Br3**. Individual types of atoms are marked with the following colors: carbon—gray, hydrogen—white, nitrogen—blue, bromine—red. Small green balls represent bond critical points, red small balls represent ring critical points, and the blue small ball represents a cage critical point.

At the end of this subsection, it is worth mentioning that, in addition to the three bond paths X⋯C${}_{\mathrm{ring}}$, there are also three bond paths for the Br⋯C${}_{\mathrm{chain}}$ contacts in the molecular graphs of **Br0**, **Br1** and **Br3**. Interestingly, there are no such bond paths for **Br2**. Apparently, the strong electron charge withdrawal by the three -CN groups is not conducive to the formation of these bond paths (Figure 6). In contrast to **Br1**, however, in **Br3**, the bond critical points for the Br⋯C${}_{\mathrm{chain}}$ interactions are very close to RCPs, which indicates topological instability. As a consequence, the bond ellipticity in **Br3** is as high as 13.9, while in **Br1** ‘only’ 2.9.

### 2.5. Energetics of the X⋯π Interaction According to IQA

Deep insight into the energetics of the X⋯π interaction can be obtained using the IQA method [32,33]. As noted in the Methodology section, taking into account the X interaction with the entire benzene ring requires summing up the respective values obtained for the individual X⋯C${}_{\mathrm{chain}}$ and X⋯C${}_{\mathrm{no}-\mathrm{chain}}$ interactions (see Equation (12)). The resulting interaction energy values and their various components are listed in Table 8.

First of all, let us note that the obtained interaction energies, both for X⋯π and its components X⋯C${}_{\mathrm{chain}}$ and X⋯C${}_{\mathrm{no}-\mathrm{chain}}$, are negative, showing that these interactions are *stabilizing*. If we take into account the unsubstituted systems, i.e., **X0**, the interaction energy increases with the size of the X atom: H (−0.048 a.u.) < F (−0.089 a.u.) < Cl (−0.130 a.u.) < Br (−0.151 a.u.). The greater part comes from the X⋯C${}_{\mathrm{chain}}$ interactions, which can probably be explained by the slightly shorter distance X⋯C${}_{\mathrm{chain}}$ than X⋯C${}_{\mathrm{no}-\mathrm{chain}}$ (Table 1).

Interestingly, according to the results obtained by the IQA method, the attachment of three substituents, either -NH${}_{2}$ or -CN, to the benzene ring leads to much stronger X⋯π interactions. However, the exceptions are derivatives with X = H and X = Br, for which the influence of -NH${}_{2}$ or -CN is negligible. The X⋯π interaction is stronger after the insertion of -NH${}_{2}$ groups. In the case of X = F, the values of the interaction energies for **F1** and **F2** are −0.223 and −0.147 a.u., respectively, while for **Cl1** and **Cl2**, the corresponding values are −0.177 and −0.151 a.u., respectively. However, when X = H or X = Br, the presence of three -CN groups gives a slightly higher interaction energy: −0.042 a.u. for **H1** vs. −0.047 a.u. for **H2** and −0.147 a.u. for **Br1** vs. −0.150 a.u. for **Br2**. It is worth noting that, in the case of **X2** (especially **F2** and **Cl2**), i.e., systems with three groups -CN, the obtained results indicate much smaller differences between the contributions from the X⋯C${}_{\mathrm{ring}}$ and X⋯C${}_{\mathrm{no}-\mathrm{ring}}$ interactions than in the case of **X1**, i.e., the molecules having three -NH${}_{2}$ groups instead. For X = F, the corresponding contributions for **F2** are −0.027 and −0.022 a.u., while for **F1** the values are −0.016 and −0.058 a.u., respectively. For X = Cl, the corresponding values are −0.027 and −0.023 a.u. for **Cl2** and −0.023 and −0.036 a.u. for **Cl1**. Clearly less differentiation of contributions in the presence of -CN substituents can be explained by a lesser differentiation of the electron charge distribution on ring carbon atoms. Conversely, the presence of three -NH${}_{2}$ groups leads to a clear alternation of electron charge between ring carbon atoms (see Table 2) and thus the observed differentiation of contributions from the X⋯C${}_{\mathrm{chain}}$ and X⋯C${}_{\mathrm{no}-\mathrm{chain}}$ contacts. The influence of the presence of three -CH${}_{3}$ groups on the X⋯π interaction energy is negligible (up to −0.006 a.u. in the case of **Br3**). More importantly, however, the direction of changes in the $E_{\mathrm{int}}$ values points to the electron-donating inductive (i.e., +*I*) action of the methyl groups.

The results presented in Table 8 clearly show that, for the unsubstituted **X0** molecules, the stabilizing effect is almost exclusively (93–101%) due to the exchange-correlation contribution ($E_{\mathrm{ee},\mathrm{xc}}$). Moreover, in some cases (**Cl0**), the electrostatic contribution ($E_{\mathrm{elst}}$) is even slightly positive. Substitution of three groups -NH${}_{2}$ to the benzene ring changes this picture, especially in the case of **F1**. In this case, the percentage share of the exchange-correlation energy in the interaction energy changes drastically from 93.1% to only 36%, which is the result of a significantly larger contribution of $E_{\mathrm{elst}}$ in comparison with $E_{\mathrm{ee},\mathrm{xc}}$ (−0.046 and −0.012 a.u., respectively) in the contribution from the F⋯C${}_{\mathrm{no}-\mathrm{chain}}$ contact (as a consequence, %$E_{\mathrm{ee},\mathrm{xc}}$ amounts to 21% only). The dominant electrostatic contribution obtained for the X⋯C${}_{\mathrm{no}-\mathrm{chain}}$ interaction can be explained by the presence of a positive charge on the C${}_{\mathrm{no}-\mathrm{chain}}$ atoms in the **F1** molecule (Table 2). A somewhat similar situation occurs in the case of **Cl1**. Compared to **Cl0**, where both Cl⋯C${}_{\mathrm{chain}}$ and Cl⋯C${}_{\mathrm{no}-\mathrm{chain}}$ interactions are practically purely covalent (according to IQA) as %$E_{\mathrm{ee},\mathrm{xc}}$ is roughly 100%, in **Cl1**, $E_{\mathrm{elst}}$ for the Cl⋯C${}_{\mathrm{no}-\mathrm{chain}}$ contact is −0.015 a.u., and as a result of which %$E_{\mathrm{ee},\mathrm{xc}}$ is reduced to 57%. As a consequence, the share of the exchange-correlation contribution in the Cl⋯π interaction is 75%. For systems with -CN groups, the %$E_{\mathrm{ee},\mathrm{xc}}$ values for X⋯C${}_{\mathrm{chain}}$ and X⋯C${}_{\mathrm{no}-\mathrm{chain}}$ are more similar and within 86–102%. Only for **F2** are these contributions much lower (56.7% and 60.9%, respectively) showing a significant influence of the electrostatic component in the F⋯C${}_{\mathrm{ring}}$ interactions.

It can be briefly concluded that the IQA method clearly indicates that the X⋯π (X = H, F, Cl, Br) interaction in all the considered iron maiden systems is stabilizing, and the exchange-correlation energy is responsible for this stabilization. Thus, according to IQA, the X⋯π interaction is almost purely covalent. The attachment of three groups either -NH${}_{2}$ or -CN to the benzene ring generally increases the stabilization energy. In the case of systems with an apical fluorine atom, the presence of these groups increases the electrostatic contribution and, in the **F1** molecule, it is predominant (64%). As could be expected, the influence of the presence of three -CH${}_{3}$ groups on the interaction energy is negligible.

**Figure 7:**
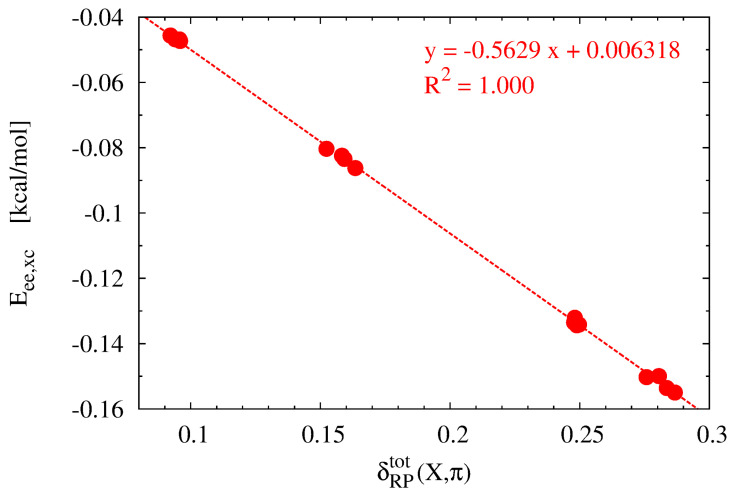
Relationship between $E_{\mathrm{ee},\mathrm{xc}}$ and $\delta_{\mathrm{RP}}^{\mathrm{tot}}$(X,π) (Equation (8)) obtained for the X⋯π interaction in the studied iron maiden molecules.

Rafat and Popelier showed that the delocalization index δ(A,B) divided by the interatomic distance A⋯B is closely related to the exchange-correlation energy of the A-B bond [72]. Therefore, it was particularly tempting to check the quality of the linear correlation between $\delta_{\mathrm{RP}}^{\mathrm{tot}}$(X,π) (Equation (8)) and the total (i.e., summed over all the X⋯C${}_{\mathrm{ring}}$ interactions) value of $E_{\mathrm{ee},\mathrm{xc}}$. The relationship between these two quantities is shown in Figure 7. As clearly seen, the linear relationship is perfect ($R^{2}$ = 1.000), which fully confirms the high linear correlation between these quantities.

### 2.6. Non-Local Analysis Based on the NCI Method

**Figure 8:**
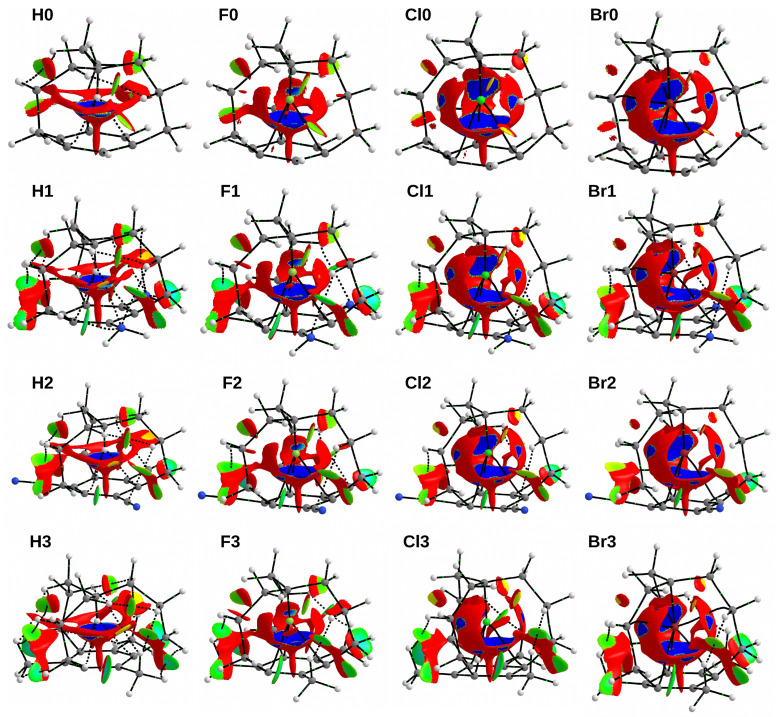
NCI-based *s*-isosurfaces (*s* = 0.5 a.u.) for the investigated **Xn** iron maiden molecules. Colors are coded according to a common $sgn(\lambda_{2})\rho$ scale (in a.u.): −0.020—blue, −0.015—cyan, −0.10—green, −0.005—yellow, and 0.000—red. Cutoff of 0.050 a.u. was used for the electron density.

As already mentioned in the Introduction section, we have recently suggested [9] that the instability of the *in* forms of molecular iron maidens is not due to the forced close contact between an apical X atom and the benzene ring, but rather to an unfavorable repulsive steric interaction between the X atom and the C-C bonds of side chains. It was interesting to see how the presence of three -NH${}_{2}$, -CN or -CH${}_{3}$ substituents affected this picture. For this purpose, the NCI method [34,35] was used, which allows for visually distinguishing weak attractive interactions from weak repulsive interactions (see Methodology). The NCI-based *s*-isosurfaces for all the investigated iron maiden molecules are shown in Figure 8.

First, however, let us recall that the proposal [9] of the dominant influence of the repulsive steric interaction between the apical X atom and the C-C bonds of the side chains has been based on the clearly visible expansion of the repulsive region between X and these C-C side chain bonds when the the size of the X atom increases: H→F→Cl→Br (Figure 8). Only in the chlorine and bromine systems are these repulsive surfaces pierced by small blue areas of attractive Cl/Br⋯C interactions. In the case of **Br0**, **Br1** and **Br3**, these interactions are further emphasized by the presence of three bond paths.

The characterization of the region between the X atom and the surface of the benzene ring is more complicated. Apart from the characteristic spindle-shaped repulsive region along the axis of the ring, a confluent funnel-shaped area of repulsion and attraction is visible above it. Therefore, the NCI method is not able to unambiguously determine the nature of the X⋯π interaction in the considered iron maiden systems; rather, both components occur simultaneously here.

The presence of three -NH${}_{2}$, -CN or -CH${}_{3}$ groups in the benzene ring only slightly influences the discussed areas of weak repulsion and weak attraction inside the iron maiden cage. Rather, the differences clearly visible through the appearance of new areas of weak repulsion and weak attraction appear on the outer sides of the substituted molecules. These regions are related to the interactions between some of the atoms of the substituted groups with some of the side chain atoms. In the case of systems with -NH${}_{2}$ groups, a weak attractive interaction between the nitrogen atom and one of the hydrogen atoms attached to the central bridge carbon atom is clearly visible. This interaction is followed by the N⋯H bond path. Nevertheless, this region merges with a region of repulsion that divides another region of weak attraction between two hydrogen atoms. In turn, this H⋯H interaction is not followed by a bond path. Quite similar areas are found in counterparts with three -CN groups. In this case, however, the first attractive region is associated with the C⋯H interaction, which may (**H2**, **F2**, **Cl2**) or may not (**Br2**) be associated with a bond path, and the second with the N⋯H interaction, which in no case is tracked by a bond path. In the case of the **X3** species, these side regions of weak attraction and weak repulsion are qualitatively identical, but this time the former are related to C-H⋯H-C interactions, where one of the C-H bonds comes from the attached methyl group and the other from the propylene side chain. Most of these interactions are related to the presence of an appropriate bond path. The lack of a significant influence of the presence of three -NH${}_{2}$, -CN or -CH${}_{3}$ groups in the benzene ring on the distribution of areas of weak repulsion and attraction inside the cage structure of iron maiden molecules confirms the already discussed rather surprising stability of their electronic structure and resistance to changes.

By the way, it is worth noting the small areas of repulsion between the C-H bonds of the carbon side chains. In systems with apical chlorine, and especially fluorine and hydrogen, this region merges seamlessly with the region of weak attraction between two hydrogen atoms. Moreover, in the **Hn** systems, the H⋯H interaction is additionally marked by the presence of a bond path. This kind of area between two hydrogen atoms can be a strong argument for the H⋯H attractive interactions in aliphatic chains. Most likely, the presence of such an area was documented for the first time in branched octane by Johnson et al. in their 2010 paper on the NCI method [34]. More recently, an attractive H⋯H region has been found between ethylene groups in ZnEt${}_{2}$ in complexes with carbenes [75].

## 3. Methodology

All calculations were performed on the ωB97X-D/6-311++G(d,p) level of theory that is utilizing the range-separated dispersion-corrected hybrid ωB97X-D exchange-correlation functional by Chai and Head-Gordon [76] and the 6-311++G(d,p) basis set [77]. It was shown that ωB97X-D is one of the best exchange-correlation functionals for general use [78]. The 6-311++G(d,p) basis set is of the triple-zeta type and contains both polarization and diffuse functions on all atoms [77,79]. The presence of diffuse functions is necessary to reliably describe the lone electron pairs on halogen and nitrogen atoms. All the presented iron maiden molecules correspond to the true minima on the potential energy surface as indicated by the lack of imaginary frequencies. Both the geometry optimization and frequency calculations were performed with the Gaussian 16 package [80].

In this article, the *in* and *out* forms of the reference molecule *in*-[3${}^{4,10}$][7]metacyclophane (**1**) (Figure 1) and its X- and ring-substituted derivatives (X = F, Cl, Br; ring substituent = -NH${}_{2}$, -CN, -CH${}_{3}$) are considered (see Figure 2). For simplicity, the compact notation **Xn** will be used from now on, where **X** = **H**, **F**, **Cl**, **Br** and **n** = **0** for the unsubstituted benzene ring, **n** = **1** for the ring with three groups -NH${}_{2}$, **n** = **2** for the ring with three groups -CN, and **n** = **3** for the ring with three groups -CH${}_{3}$. Thus, the parent molecule **1** is now on **H0** and, e.g., its derivative with an apical chlorine atom and three -NH${}_{2}$ groups in the benzene ring is **Cl1**. As already mentioned, the considered molecules have C${}_{3}$ symmetry.

To consider the energetics of the *out*→*in* isomerization, the energy of this transition was determined, $\Delta E_{in-out}$ = $E_{\mathrm{tot}}$(**Xn**${}^{in}$) −$E_{\mathrm{tot}}$(**Xn**${}^{out}$), where $E_{\mathrm{tot}}$(**Xn**${}^{in}$) and $E_{\mathrm{tot}}$(**Xn**${}^{out}$) are the total energies of the *in* and *out* forms, respectively. In addition, in order to study the effect of the H → X (X = F, Cl, Br) substitution, the energy of the X substituent (converted to kcal/mol) in the **Xn**${}^{in/out}$ molecule, $\Delta E_{\mathrm{Xn}}^{in/out}$(X), was determined using the following equations [50]:

(1) $$E_{\mathrm{MeX}}\left(X\right)=E\left(\mathrm{MeX}\right)-E\left({\mathrm{CH}}_{4}\right)<0,$$

(2) $$E_{{\mathrm{Xn}}^{\mathrm{in}/\mathrm{out}}}\left(X\right)=E\left({\mathrm{Xn}}^{\mathrm{in}/\mathrm{out}}\right)-E\left({\mathrm{Hn}}^{\mathrm{in}/\mathrm{out}}\right)<0,$$

(3) $$\Delta E_{\mathrm{Xn}}^{\mathrm{in}/\mathrm{out}}\left(X\right)=627.5095\cdot \left[E_{{\mathrm{Xn}}^{\mathrm{in}/\mathrm{out}}}\left(X\right)-E_{\mathrm{MeX}}\left(X\right)\right],$$

where the symbol without a subscript is the total energy of the molecule shown in parantheses. For example, *E*(MeX) is the total energy of halogenomethane and *E*(**Xn**${}^{\mathrm{in}/\mathrm{out}}$) is the total energy of either the *in* or *out* form of the **Xn** molecule. Importantly, $\Delta E_{\mathrm{Xn}}^{\mathrm{in}/\mathrm{out}}\left(X\right)$ is actually the energy of the following homodesmotic reaction, as can be seen by substituting the right-hand sides of Equations (1) and (2) into Equation (3):

(4) $$\mathrm{Hn}+\mathrm{MeX}⇌\mathrm{Xn}+{\mathrm{CH}}_{4}.$$

As a consequence, the substituent energy $\Delta E_{\mathrm{Xn}}^{\mathrm{in}/\mathrm{out}}$(X) has a clear physical meaning. Namely, the value of $\Delta E_{\mathrm{Xn}}^{\mathrm{in}/\mathrm{out}}$(X) tells how much X prefers to be (if $\Delta E_{\mathrm{Xn}}^{\mathrm{in}/\mathrm{out}}$(X) <0) or not (if $\Delta E_{\mathrm{Xn}}^{\mathrm{in}/\mathrm{out}}$(X) >0) in the **Xn** molecule compared to MeX. Halogenomethane was chosen as a reference because it preserves the formal sp${}^{3}$ hybridization on the keystone carbon atom, and it is also the simplest molecule. However, this does not matter much, since the intention was to obtain relative energies, not absolute ones.

One way to estimate the bond strength is to determine its order [81]. This was completed by computing the very popular but quite outdated Wiberg Bond Index (WBI) [20]:

(5) $${\mathrm{WBI}}_{\mathrm{AB}}=\sum_{\alpha \in A}\sum_{\beta \in B}P_{\alpha \beta }^{2}$$

and its more recent generalization in the form of the Mayer Bond Order (MBO) [16,17,18,19]:

(6) $${\mathrm{MBO}}_{\mathrm{AB}}=\sum_{\alpha \in A}\sum_{\beta \in B}{\left(\mathrm{PS}\right)}_{\alpha \beta }{\left(\mathrm{PS}\right)}_{\beta \alpha }$$

where **P** and **S** are the density and atomic orbital overlap matrices, respectively. Unlike WBI, MBO can also take negative values.

Bader’s QTAIM is an important and frequently used theoretical tool to describe various types of inter- and intramolecular interactions [21,22,23,24,25]. In particular, it is helpful to know the electron density (ρ), its Laplacian ($\nabla^{2}\rho =\lambda_{1}+\lambda_{2}+\lambda_{3}$, where $\lambda_{i}$ are the eigenvalues of the Hessian matrix of the electron density), the total electronic energy density (*H*) [82] and the bond ellipticity ($\varepsilon =\lambda_{1}/\lambda_{2}-1$ where $|\lambda_{1}\left|\;\geq \;\right|\lambda_{2}|$) at the bond critical point (BCP) [21] of interest. The value of *H* is negative for interactions with significant sharing of electrons and therefore is a measure of the covalence of an interaction, while the positive value of *H* is characteristic for non-covalent interactions such as, e.g., weak hydrogen bonds. The bond ellipticity is a measure of the anisotropy in the electron density distribution at a critical point [21]. Originally, it was used to describe the π-electron nature of multiple bonds; nevertheless, it is also often used to describe charge delocalization [83] and molecular strain and instability [84]. As mentioned in the Introduction, the analyzed iron maiden molecules are characterized by the presence of three equivalent (due to C${}_{3}$ symmetry) bond paths (BP) [21] X⋯C${}_{\mathrm{ring}}$. Each of them traces the maximum electron density value relative to neighboring points and has a minimum in the BCP between the linked nuclei (attractors). These QTAIM parameters, i.e., ρ, $\nabla^{2}\rho$, *H* and ε, were calculated in BCP${}_{X\cdots C_{\mathrm{ring}}}$. However, another valuable QTAIM-based parameter is the A-B bond delocalization index, δ(A,B). It describes the average number of electrons delocalized (shared) between atoms A and B and becomes a bond index when atoms A and B are connected to each other by a bond path [21,85,86,87,88,89]. Moreover, δ(A,B) divided by the distance A⋯B is closely related to the exchange-correlation energy of the A-B bond [72]. Although, as we have shown [9], the X⋯π interaction may well be characterized by a single value of δ(X,C_ring_), the total delocalization index [73,74] was also determined to take into account the fact that X actually interacts with the entire ring, i.e., with all six of its carbon atoms, not just one:

(7) $$\delta^{\mathrm{tot}}\left(X,\pi \right)=\sum_{i=1}^{6}\delta \left(X,C_{\mathrm{ring}}^{i}\right)=3\delta \left(X,C_{\mathrm{ring}}^{\mathrm{chain}}\right)+3\delta \left(X,C_{\mathrm{ring}}^{\mathrm{no}-\mathrm{chain}}\right).$$

(8) $$\delta_{\mathrm{RP}}^{\mathrm{tot}}\left(X,\pi \right)=\sum_{i=1}^{6}\frac{\delta (X,C_{\mathrm{ring}}^{i})}{d_{X\cdots C_{\mathrm{ring}}^{i}}}=3\frac{\delta (X,C_{\mathrm{ring}}^{\mathrm{chain}})}{d_{X\cdots C_{\mathrm{ring}}^{\mathrm{chain}}}}+3\frac{\delta (X,C_{\mathrm{ring}}^{\mathrm{no}-\mathrm{chain}})}{d_{X\cdots C_{\mathrm{ring}}^{\mathrm{no}-\mathrm{chain}}}}.$$

The IQA method [32,33] was used to gain an in-depth insight into the energetics of the X⋯π interaction. It allows for a decomposition of the total energy of a system into mono- and polyatomic contributions. Of the many IQA parameters available, the interatomic interaction energy is most likely the most useful:

(9) $$E_{\mathrm{int}}^{E_{1}E_{2}}=E_{\mathrm{nn}}^{E_{1}E_{2}}+E_{\mathrm{ne}}^{E_{1}E_{2}}+E_{\mathrm{en}}^{E_{1}E_{2}}+E_{\mathrm{ee}}^{E_{1}E_{2}}\;\;\;\;\;\;\left(E_{1}\neq E_{2}\right),$$

In this equation, $E_{\mathrm{nn}}^{E_{1}E_{2}}$ is the repulsion energy between nuclei of atoms E${}_{1}$ and E${}_{2}$, $V_{\mathrm{ne}}^{E_{1}E_{2}}$ is the attraction energy between the nucleus of the atom E${}_{1}$ and the electrons of the atom E${}_{2}$, $E_{\mathrm{en}}^{E_{1}E_{2}}$ is the attraction energy between electrons of the atom E${}_{1}$ and the nucleus of the atom E${}_{2}$ and $E_{\mathrm{ee}}^{E_{1}E_{2}}$ is the interatomic two-electron repulsion energy. The sum of the middle two terms gives the energy of the interatomic nucleus–electron attraction ($E_{\mathrm{neen}}^{E_{1}E_{2}}$). Then, the interelectron repulsion energy can be further divided into a sum of the purely classical (Coulombic) contribution and the exchange-correlation (i.e., the non-classical term) energy:

(10) $$E_{\mathrm{ee}}^{E_{1}E_{2}}=E_{\mathrm{ee},C}^{E_{1}E_{2}}+E_{\mathrm{ee},\mathrm{xc}}^{E_{1}E_{2}}$$

Moreover, the sum of the first three terms in Equation (9) and $E_{\mathrm{ee},C}^{E_{1}E_{2}}$ gives the electrostatic energy, leading to a compact expression for the interatomic interaction energy:

(11) $$E_{\mathrm{int}}^{E_{1}E_{2}}=E_{\mathrm{elst}}^{E_{1}E_{2}}+E_{\mathrm{ee},\mathrm{xc}}^{E_{1}E_{2}}$$

In this way, the interatomic interaction energy $E_{\mathrm{int}}^{E_{1}E_{2}}$ is divided into its classical electrostatic contribution and a non-classical exchange-correlation contribution. An important ability of the IQA approach, not to be underestimated, is that $E_{\mathrm{int}}^{E_{1}E_{2}}$ can be computed for any pair of E${}_{1}$ and E${}_{2}$ atoms and not necessarily linked to each other by a bond path. Moreover, IQA does not require any reference system or any further model-dependent assumptions (as is the case, for example, in ETS-NOCV [36,37,38,39], where results depend on the system defragmentation scheme). To take into account the fact that the apical X atom interacts with the entire benzene ring and that there are two types of ring carbon atoms (chain, no-chain), as in the case of the total delocalization index (Equations (7) and (8)), the interaction energy and its components for the X⋯π interaction were determined by summing up all the energy components computed for the individual X⋯C${}_{\mathrm{ring}}$ contacts:

(12) $$E_{\mathrm{int}}^{X\cdots \pi }=\sum_{i=1}^{6}E_{\mathrm{int}}^{X\cdots C_{\mathrm{ring}}^{i}}=3E_{\mathrm{int}}^{X\cdots C_{\mathrm{ring}}^{\mathrm{chain}}}+3E_{\mathrm{int}}^{X\cdots C_{\mathrm{ring}}^{\mathrm{no}-\mathrm{chain}}}$$

In order to obtain a non-local insight into the characteristics of the interaction between the apical X atom and the remaining atoms, especially of the benzene ring, the NCI method was used [34,35]. This method is based on the reduced electron density gradient ($s=1/\left(2{\left(3\pi^{2}\right)}^{1/3}\right)\left|\nabla \rho \right|/\rho^{4/3}$) and $sgn(\lambda_{2})\rho$, i.e., the electron density multiplied by the sign of the second eigenvalue of the electron density Hessian matrix ($\lambda_{2}$). As a consequence, NCI allows for displaying individual weak interactions as certain regions of real space rather than as local features of a BCP corresponding to a pairwise interatomic contact. Most importantly, these interactions can be easily and visually (by using different colors) separated into attractive (if $\lambda_{2}<0$) and repulsive (if $\lambda_{2}>0$) [34,35]. The QTAIM-, IQA- and NCI-based calculations were performed using the AIMAll program [90].

## 4. Conclusions

The so-called molecular iron maidens are interesting examples of cyclophanes distinguished by the unique ultrashort contact between an apical hydrogen atom or its small substitute and the benzene ring. It is widely believed that this forced ultrashort contact X⋯π is associated with a large spatial hindrance giving iron maidens specific properties. It seems, therefore, that these properties should strongly depend on the electronic features of the benzene ring. The main aim of the article was to investigate the influence of a strong charge enrichment or depletion of this ring on the properties of iron maiden molecules, especially on the characteristics of the ultrashort C-X⋯π contact. For this purpose, either three -NH${}_{2}$ groups characterized by extremely strong electron-donating properties or three -CN groups characterized by extremely strong electron-accepting properties were substituted into the benzene ring of *in*-[3${}^{4,10}$][7]metacyclophane and its halogen derivatives (X = F, Cl, Br). In addition, their counterparts with three -CH${}_{3}$ groups, characterized almost only by the inductive effect, were also tested. Surprisingly, it has been shown that, despite such extremely strong electronic properties of both the -NH${}_{2}$ or -CN substituents, the tested iron maiden molecules show quite high resistance to their presence in the benzene ring.

In the case of halogenated derivatives, i.e., when X = halogen, the presence of three -NH${}_{2}$, -CN or -CH${}_{3}$ groups slightly reduces the unfavorable endothermic effect of the *out*→*in* isomerization process. The energy of this process strongly depends on the X substituent. In the case of systems with either -NH${}_{2}$ or -CN, the negative values of Mayer Bond Order suggest the anti-bonding nature of the X⋯π interactions in the substituted iron maidens regardless of the type of X. However, in the case of unsubstituted and methyl-trisubstituted derivatives with Br, positive values suggest bonding nature of the Br⋯π interaction in these systems, which can be explained by the presence of an extremely pronounced σ-hole on the bromine atom. On the contrary, the IQA-based analysis has shown that the X⋯π interactions are stabilizing, and this stabilization increases after the insertion of either -NH${}_{2}$ or -CN groups. Although these interactions are almost purely covalent in nature according to IQA, the presence of three -NH${}_{2}$ groups on the benzene ring of the F-derivative significantly reduces the percentage contribution of the exchange-correlation energy due to the significant electrostatic interaction between the charge-depleted ring carbon atom and the F atom. The NCI-based analysis has shown that the substitution of three -NH${}_{2}$, -CN or -CH${}_{3}$ groups into the benzene ring does not significantly affect the areas of weak interactions or weak attractions within the cage structure of iron maiden molecules. The new regions, on the other hand, are observed on the periphery of molecules and are associated with interactions between substituents and side chain atoms.

## Figures and Tables

**Table 1:** The most important geometric parameters characterizing *in* forms of the **Xn** iron maiden molecules (see Figure 3). Bond lengths in Å, plane angles in degrees.

Xn	$d_{\mathrm{CX}}$	$\Delta d_{\mathrm{CX}}^{\mathrm{MeX}}$ ^*a*^	$\Delta d_{\mathrm{CX}}^{\mathrm{out}}$ ^*b*^	$d_{X\cdots \pi }$ ^*c*^	$d_{X\cdots \mathrm{RCP}}$ ^*d*^	$d_{C\cdots \pi }$ ^*e*^	$d_{X\cdots C}$ ^*f*^	Fold ^*g*^	$d_{\mathrm{Ar}}$ ^*h*^	$\alpha_{1}$ ^*i*^	$\alpha_{2}$ ^*i*^	$\alpha_{3}$ ^*i*^	$\alpha_{\mathrm{CCX}}$ ^*i*^
**H0**	1.057	−0.033	−0.033	1.667	1.598	2.724	2.152/2.193	0.041	2.787	109.9	118.7	115.7	103.8
**H1**	1.060	−0.031	−0.031	1.679	1.596	2.739	2.174/2.204	0.030	2.808	111.2	118.2	115.5	104.0
**H2**	1.055	−0.035	−0.033	1.667	1.606	2.723	2.152/2.204	0.053	2.802	109.4	119.6	115.9	103.4
**H3**	1.058	−0.033	−0.033	1.662	1.594	2.719	2.146/2.202	0.057	2.804	110.2	119.4	116.0	103.9
**F0**	1.345	−0.040	−0.103	1.919	1.849	3.264	2.335/2.410	0.075	2.792	115.3	125.0	112.8	99.4
**F1**	1.348	−0.037	−0.102	1.924	1.847	3.273	2.365/2.405	0.040	2.817	116.7	124.1	112.6	99.7
**F2**	1.355	−0.030	−0.087	1.894	1.829	3.249	2.306/2.409	0.103	2.807	114.7	125.4	113.2	99.2
**F3**	1.344	−0.042	−0.106	1.914	1.847	3.258	2.333/2.415	0.081	2.809	115.8	126.1	113.1	99.3
**Cl0**	1.654	−0.136	−0.234	2.073	1.984	3.728	2.465/2.546	0.081	2.815	121.0	131.2	114.8	94.9
**Cl1**	1.661	−0.130	−0.233	2.061	1.954	3.722	2.486/2.524	0.038	2.849	122.6	130.3	114.7	95.5
**Cl2**	1.655	−0.135	−0.226	2.068	1.973	3.723	2.442/2.567	0.126	2.829	120.4	131.0	115.3	94.8
**Cl3**	1.657	−0.134	−0.231	2.068	1.972	3.725	2.463/2.548	0.085	2.832	121.7	132.5	115.1	95.1
**Br0**	1.774	−0.168	−0.282	2.122	2.026	3.895	2.513/2.584	0.070	2.824	122.7	133.9	116.7	92.8
**Br1**	1.782	−0.159	−0.280	2.100	1.978	3.882	2.526/2.559	0.032	2.865	124.1	133.0	116.5	93.5
**Br2**	1.770	−0.172	−0.278	2.124	2.020	3.894	2.490/2.617	0.128	2.838	122.2	133.2	117.3	92.8
**Br3**	1.777	−0.164	−0.277	2.112	2.007	3.889	2.506/2.584	0.078	2.842	123.2	135.0	117.0	93.1

^*a*^ Change of the CX bond length relative to methane or halogenomethane. ^*b*^ Change of the CX bond length relative to the *out* form. ^*c*^ The distance between X and the centroid of the benzene ring obtained as 1/2(*H*^*chain*^ + *H*^*no-chain*^) where *H*^*chain/no-chain*^ = $\sqrt{({d_{X\cdots C})}^{2}-1/3({d_{C\cdots C})}^{2}}$ with C being *either* the chain or no-chain carbon atoms of the ring. ^*d*^ The distance between X and RCP of the benzene ring. ^*e*^ he distance between the keystone C and the centroid of the benzene ring obtained as *d*_X⋯π_ + *d*_CX_. ^*f*^ The distance between X and the chain/non-chain C atom of the benzene ring. ^*g*^ The folding of the benzene ring understood as $d_{X\cdots C}^{\mathrm{no}-\mathrm{chain}}-d_{X\cdots C}^{\mathrm{chain}}$. ^*h*^ The size of the benzene ring understood as the distance between opposite carbon atoms. ^*i*^ See Figure 3 for the meanings of the respective angles.

**Table 2:** Electron charge distribution within the benzene ring in **Xn** molecules: electron density in RCP ($\rho_{\mathrm{RCP}}$), Hirshfeld atomic charges on chain and no-chain C atom ($q_{C}^{\mathrm{chain}}$ and $q_{C}^{\mathrm{no}-\mathrm{chain}}$, respectively), total charge on carbon atoms of the benzene ring ($Q_{C}^{\mathrm{ring}}$). Atomic units are used.

Xn	$\rho_{\mathrm{RCP}}$	$q_{C}^{\mathrm{chain}}$	$q_{C}^{\mathrm{no}-\mathrm{chain}}$	$Q_{C}^{\mathrm{ring}}$	Xn	$\rho_{\mathrm{RCP}}$	$q_{C}^{\mathrm{chain}}$	$q_{C}^{\mathrm{no}-\mathrm{chain}}$	$Q_{C}^{\mathrm{ring}}$
**H0**	0.024	0.005	−0.051	−0.139	**F0**	0.024	−0.010	−0.066	−0.229
**H1**	0.022	−0.067	0.045	−0.066	**F1**	0.022	−0.094	0.041	−0.160
**H2**	0.022	0.049	0.007	0.169	**F2**	0.022	0.044	−0.010	0.103
**H3**	0.023	−0.011	−0.002	−0.038	**F3**	0.022	−0.027	−0.012	−0.118
**Cl0**	0.024	−0.015	−0.059	−0.220	**Br0**	0.023	−0.019	−0.050	−0.206
**Cl1**	0.022	−0.113	0.048	−0.193	**Br1**	0.021	−0.119	0.054	−0.196
**Cl2**	0.022	0.041	−0.010	0.095	**Br2**	0.022	0.040	−0.007	0.098
**Cl3**	0.023	−0.035	−0.004	−0.117	**Br3**	0.022	−0.038	0.002	−0.106

**Table 3:** The influence of the presence of three substituents in the benzene ring of the considered iron maiden molecules on the values of geometric parameters: ⇑—systematic increase, ↑c—constant increase, ↓—unsystematic decrease, ↓c—constant decrease, ∼ X—increase or decrease depending on X.

Substituent	$d_{\mathrm{CX}}$	$d_{X\cdots \pi }$	$d_{C\cdots \pi }$	Fold	$d_{\mathrm{Ar}}$	$\alpha_{1}$	$\alpha_{2}$	$\alpha_{3}$	$\alpha_{\mathrm{CCX}}$
-NH${}_{2}$	⇑	∼ X	∼ X	↓	⇑	↑c	↓	↓c	⇑
-CN	∼ X	∼ X	↓	⇑	↑c	↓c	∼ X	⇑	↓
-CH${}_{3}$	∼ X	↓	↓	↑	↑c	↑	↑	↑c	∼ X

**Table 4:** The *out* → *in* isomerization energies (in kcal/mol) for the considered iron maiden molecules.

**Xn**	X			
	H	F	Cl	Br
**X0**	−12.3 (ref.)	50.5 (ref.)	171.1 (ref.)	229.9 (ref.)
**X1**	−12.8 (−0.5)	47.7 (−2.8)	160.8 (−10.3)	216.1 (−13.8)
**X2**	−10.6 (1.7)	44.3 (−6.2)	167.3 (−3.8)	228.1 (−1.8)
**X3**	−13.1 (−0.8)	51.4 (+0.9)	167.9 (−3.2)	224.1 (−5.8)

**Table 5:** The X-substituent energies (in kcal/mol) for *in* and *out* forms of the **Xn** iron maiden molecules.

**Xn**	X					
	F		Cl		Br	
	*in*	*out*	*in*	*out*	*in*	*out*
**X0**	48.6 (ref.)	−14.2 (ref.)	179.2 (ref.)	−4.2 (ref.)	240.6 (ref.)	−1.6 (ref.)
**X1**	45.1 (−3.5)	−15.4 (−1.2)	167.6 (−11.6)	−6.0 (−1.8)	225.3 (−15.3)	−3.5 (−1.9)
**X2**	42.3 (−6.3)	−12.5 (1.7)	176.1 (−3.1)	−1.7 (2.5)	239.7 (−0.9)	1.0 (2.6)
**X3**	49.8 (+1.2)	−14.7 (−0.5)	176.0 (−3.2)	−5.0 (−0.8)	234.8 (−5.8)	−2.5 (−0.9)

**Table 6:** Values of Wiberg Bond Index (WBI) and Mayer Bond Order (MBO) for the X⋯C${}_{\mathrm{ring}}^{\mathrm{chain}}$ and X⋯C${}_{\mathrm{ring}}^{\mathrm{no}-\mathrm{chain}}$ interactions as well as the total WBI/MBO values obtained by summing over all X⋯C${}_{\mathrm{ring}}^{\mathrm{chain}/\mathrm{no}-\mathrm{chain}}$ interactions (X⋯π).

Xn	WBI			MBO		
	X⋯C${}_{\mathrm{ring}}^{\mathrm{chain}}$	X⋯C${}_{\mathrm{ring}}^{\mathrm{no}-\mathrm{chain}}$	X⋯π	X⋯C${}_{\mathrm{ring}}^{\mathrm{chain}}$	X⋯C${}_{\mathrm{ring}}^{\mathrm{no}-\mathrm{chain}}$	X⋯π
**H0**	0.004	0.003	0.023	−0.182	0.137	−0.136
**H1**	0.004	0.004	0.023	−0.009	−0.183	−0.575
**H2**	0.004	0.003	0.020	−0.003	−0.060	−0.190
**H3**	0.004	0.003	0.021	−0.169	0.012	−0.472
**F0**	0.010	0.009	0.057	−0.103	−0.011	−0.342
**F1**	0.012	0.010	0.067	−0.118	0.073	−0.135
**F2**	0.012	0.010	0.065	−0.089	0.052	−0.112
**F3**	0.011	0.009	0.060	−0.078	−0.004	−0.246
**Cl0**	0.014	0.012	0.076	0.032	−0.075	−0.127
**Cl1**	0.019	0.016	0.103	0.124	−0.413	−0.866
**Cl2**	0.020	0.012	0.094	−0.021	−0.784	−2.415
**Cl3**	0.015	0.012	0.081	−0.141	−0.103	−0.733
**Br0**	0.017	0.016	0.098	0.100	−0.072	0.083
**Br1**	0.024	0.025	0.145	0.372	−0.427	−0.163
**Br2**	0.024	0.014	0.111	0.685	−0.849	−0.494
**Br3**	0.019	0.017	0.107	0.198	−0.166	0.095

**Table 7:** QTAIM-based parameters for the **Xn** iron maiden molecules.

Xn	$\rho_{X\cdots C}$	$\nabla^{2}\rho_{X\cdots C}$	$H_{X\cdots C}$	$\lambda_{1}$	$\lambda_{2}$	$\varepsilon_{X\cdots C}$	$\lambda_{2}^{\mathrm{RCP}}$	$\delta^{\mathrm{tot}}$(X,π)	$\delta_{\mathrm{RP}}^{\mathrm{tot}}$(X,π)
**H0**	0.024	0.093	0.003	−0.0232	−0.0019	11.1	0.0015	0.208	0.096
**H1**	0.024	0.090	0.002	−0.0229	−0.0049	3.7	0.0037	0.202	0.092
**H2**	0.024	0.092	0.003	−0.0229	−0.0013	16.2	0.0011	0.205	0.094
**H3**	0.024	0.092	0.002	−0.0236	−0.0035	5.7	0.0026	0.208	0.096
**F0**	0.030	0.156	0.004	−0.0296	−0.0045	5.7	0.0029	0.376	0.158
**F1**	0.030	0.153	0.004	−0.0292	−0.0065	3.5	0.0041	0.363	0.152
**F2**	0.031	0.158	0.003	−0.0305	−0.0051	4.9	0.0034	0.385	0.163
**F3**	0.031	0.156	0.004	−0.0303	−0.0058	4.2	0.0040	0.378	0.159
**Cl0**	0.039	0.149	−0.001	−0.0330	−0.0075	3.4	0.0030	0.621	0.248
**Cl1**	0.040	0.148	−0.001	−0.0341	−0.0098	2.5	0.0041	0.620	0.248
**Cl2**	0.040	0.146	−0.002	−0.0332	−0.0105	2.2	0.0057	0.622	0.249
**Cl3**	0.040	0.147	−0.001	−0.0346	−0.0087	3.0	0.0043	0.625	0.250
**Br0**	0.041	0.141	−0.003	−0.0333	−0.0066	4.0	0.0006	0.714	0.281
**Br1**	0.043	0.139	−0.003	−0.0353	−0.0101	2.5	0.0025	0.728	0.287
**Br2**	0.042	0.136	−0.004	−0.0335	−0.0117	1.9	0.0054	0.702	0.276
**Br3**	0.042	0.139	−0.003	−0.0355	−0.0086	3.1	0.0027	0.721	0.284

**Table 8:** IQA-based energy (in a.u.) terms (see Methodology) computed for the X⋯C${}_{\mathrm{chain}}$, X⋯C${}_{\mathrm{no}-\mathrm{chain}}$ and X⋯π interactions in the **Xn** iron maiden molecules.

Xn	Contact	$E_{\mathrm{neen}}$	$E_{\mathrm{nn}}$	$E_{\mathrm{ee}}$	$E_{\mathrm{ee},C}$	$E_{\mathrm{ee},\mathrm{xc}}$	$\%E_{\mathrm{ee},\mathrm{xc}}$	$E_{\mathrm{elst}}$	$E_{\mathrm{int}}$
**H0**	C${}_{\mathrm{chain}}$	−2.929	1.476	1.445	1.453	−0.008	98.8	0.000	−0.008
	C${}_{\mathrm{no}-\mathrm{chain}}$	−2.882	1.448	1.426	1.433	−0.008	98.0	0.000	−0.008
	π (sum)	−17.430	8.771	8.612	8.659	−0.047	98.4	−0.001	**−0.048**
**H1**	C${}_{\mathrm{chain}}$	−2.898	1.460	1.430	1.438	−0.008	99.2	0.000	−0.008
	C${}_{\mathrm{no}-\mathrm{chain}}$	−2.787	1.441	1.340	1.347	−0.007	120.3	0.001	−0.006
	π (sum)	−17.054	8.704	8.308	8.354	−0.046	107.9	0.003	**−0.042**
**H2**	C${}_{\mathrm{chain}}$	−2.964	1.464	1.492	1.500	−0.008	98.0	0.000	−0.008
	C${}_{\mathrm{no}-\mathrm{chain}}$	−2.921	1.441	1.473	1.481	−0.008	100.0	0.000	−0.008
	π (sum)	−17.656	8.714	8.895	8.942	−0.047	99.0	0.000	**−0.047**
**H3**	C${}_{\mathrm{chain}}$	−2.931	1.480	1.442	1.451	−0.008	97.5	0.000	−0.009
	C${}_{\mathrm{no}-\mathrm{chain}}$	−2.858	1.442	1.408	1.416	−0.007	97.3	0.000	−0.008
	π (sum)	−17.364	8.764	8.552	8.599	−0.047	97.4	−0.001	**−0.049**
**F0**	C${}_{\mathrm{chain}}$	−25.276	12.239	13.018	13.032	−0.015	78.2	−0.004	−0.019
	C${}_{\mathrm{no}-\mathrm{chain}}$	−24.570	11.856	12.703	12.715	−0.013	119.3	0.002	−0.011
	π (sum)	−149.536	72.287	77.161	77.243	−0.082	93.1	−0.006	**−0.089**
**F1**	C${}_{\mathrm{chain}}$	−24.985	12.085	12.884	12.899	−0.015	91.0	−0.001	−0.016
	C${}_{\mathrm{no}-\mathrm{chain}}$	−23.953	11.883	12.012	12.025	−0.012	20.9	−0.046	−0.058
	π (sum)	−146.816	71.902	74.690	74.771	−0.080	36.0	−0.143	**−0.223**
**F2**	C${}_{\mathrm{chain}}$	−25.498	12.394	13.077	13.093	−0.015	56.7	−0.012	−0.027
	C${}_{\mathrm{no}-\mathrm{chain}}$	−24.461	11.864	12.576	12.589	−0.013	60.9	−0.008	−0.022
	π (sum)	−149.879	72.774	76.958	77.044	−0.086	58.6	−0.061	**−0.147**
**F3**	C${}_{\mathrm{chain}}$	−25.318	12.248	13.053	13.068	−0.015	87.9	−0.002	−0.017
	C${}_{\mathrm{no}-\mathrm{chain}}$	−24.512	11.835	12.666	12.679	−0.013	109.9	0.001	−0.011
	π (sum)	−149.491	72.248	77.157	77.240	−0.083	96.7	−0.003	**−0.086**
**Cl0**	C${}_{\mathrm{chain}}$	−43.979	21.898	22.057	22.080	−0.024	97.9	0.000	−0.024
	C${}_{\mathrm{no}-\mathrm{chain}}$	−42.685	21.202	21.464	21.485	−0.021	105.5	0.001	−0.019
	π (sum)	−259.993	129.299	130.563	130.695	−0.132	101.3	0.002	**−0.130**
**Cl1**	C${}_{\mathrm{chain}}$	−43.678	21.709	21.946	21.970	−0.024	103.0	0.001	−0.023
	C${}_{\mathrm{no}-\mathrm{chain}}$	−41.699	21.382	20.282	20.302	−0.020	57.3	−0.015	−0.036
	π (sum)	−256.132	129.273	126.682	126.816	−0.134	75.5	−0.043	**−0.177**
**Cl2**	C${}_{\mathrm{chain}}$	−44.280	22.107	22.146	22.171	−0.025	91.3	−0.002	−0.027
	C${}_{\mathrm{no}-\mathrm{chain}}$	−42.117	21.023	21.071	21.090	−0.020	85.9	−0.003	−0.023
	π (sum)	−259.191	129.391	129.649	129.783	−0.134	88.9	−0.017	**−0.151**
**Cl3**	C${}_{\mathrm{chain}}$	−44.069	21.914	22.130	22.155	−0.024	100.5	0.000	−0.024
	C${}_{\mathrm{no}-\mathrm{chain}}$	−42.604	21.180	21.404	21.424	−0.020	102.0	0.000	−0.020
	π (sum)	−260.018	129.284	130.601	130.735	−0.134	101.2	0.002	**−0.133**
**Br0**	C${}_{\mathrm{chain}}$	−88.333	44.214	44.093	44.119	−0.026	98.5	0.000	−0.026
	C${}_{\mathrm{no}-\mathrm{chain}}$	−86.108	43.012	43.072	43.012	−0.024	99.7	0.000	−0.024
	π (sum)	−523.322	261.494	261.494	261.677	−0.150	99.1	−0.001	**−0.151**
**Br1**	C${}_{\mathrm{chain}}$	−88.093	43.992	44.072	44.100	−0.028	97.7	−0.001	−0.028
	C${}_{\mathrm{no}-\mathrm{chain}}$	−84.097	43.434	40.641	44.665	−0.024	115.2	0.003	−0.021
	π (sum)	−516.569	262.280	254.141	254.296	−0.155	105.1	0.008	**−0.147**
**Br2**	C${}_{\mathrm{chain}}$	−88.912	44.636	44.249	44.277	−0.028	101.8	0.001	−0.028
	C${}_{\mathrm{no}-\mathrm{chain}}$	−84.501	42.456	42.022	42.044	−0.022	97.9	0.000	−0.022
	π (sum)	−520.240	261.276	258.814	258.964	−0.150	100.1	0.000	**−0.150**
**Br3**	C${}_{\mathrm{chain}}$	−88.742	44.348	44.366	44.393	−0.028	97.3	−0.001	−0.028
	C${}_{\mathrm{no}-\mathrm{chain}}$	−86.015	43.005	42.987	43.010	−0.024	99.0	0.000	−0.024
	π (sum)	−524.272	262.058	262.057	262.211	−0.154	98.1	−0.003	**−0.157**

## Data Availability

Data available from the author on reasonable request.

## Abbreviations

The following abbreviations are used in this manuscript:

WBI	Wiberg Bond Index
MBO	Mayer Bond Order
QTAIM	Quantum Theory of Atoms in Molecules
BCP	Bond Critical Point
RCP	Ring Critical Point
BP	Bond Path
IQA	Interacting Quantum Atoms
NCI	Noncovalent Interaction Index
ETS-NOCV	Extended Transition State with the Natural Orbitals for Chemical Valence Method
SAPT	Symmetry-Adapted Perturbation Theory
sEDA	sigma-Electron-Donor-Acceptor
